# Cardiac radiation dose predicts survival in esophageal squamous cell carcinoma treated by definitive concurrent chemotherapy and intensity modulated radiotherapy

**DOI:** 10.1186/s13014-020-01664-7

**Published:** 2020-09-22

**Authors:** Tzu-Hui Pao, Wei-Lun Chang, Nai-Jung Chiang, Jeffrey Shu-Ming Chang, Chia-Ying Lin, Wu-Wei Lai, Yau-Lin Tseng, Yi-Ting Yen, Ta-Jung Chung, Forn-Chia Lin

**Affiliations:** 1 Department of Radiation Oncology, National Cheng Kung University Hospital, College of Medicine, National Cheng Kung University, No.138, Sheng Li Road, Tainan, 70456 Taiwan; 2 Department of Internal Medicine, National Cheng Kung University Hospital, College of Medicine, National Cheng Kung University, Tainan, Taiwan; 3 National Institute of Cancer Research, National Health Research Institutes, Tainan, Taiwan; 4 Department of Diagnostic Radiology, National Cheng Kung University Hospital, College of Medicine, National Cheng Kung University, Tainan, Taiwan; 5 Department of Surgery, National Cheng Kung University Hospital, College of Medicine, National Cheng Kung University, Tainan, Taiwan

**Keywords:** Esophageal cancer, Chemoradiotherapy, Intensity modulated radiotherapy, Cardiac radiation dose

## Abstract

**Background:**

The prognostic significance of cardiac radiation dose in esophageal cancer after definitive concurrent chemoradiotherapy (CCRT) remains largely unknown. We aimed to investigate the association between cardiac dose-volume parameters and overall survival (OS) in esophageal squamous cell carcinoma (ESCC) after definitive CCRT.

**Methods:**

One hundred and twenty-one ESCC patients undergoing definitive CCRT with intensity modulated radiotherapy technique between 2008 and 2018 were reviewed. Cardiac dose-volume parameters were calculated. Survival of patients and cumulative incidence of adverse events were estimated by the Kaplan–Meier method and compared between groups by the log-rank test. The prognostic significance of cardiac dose-volume parameters was determined with multivariate Cox proportional hazards regression analysis.

**Results:**

Median follow-up was 16.2 months (range, 4.3–109.3). Median OS was 18.4 months. Heart V5, V10, and V20 were independent prognostic factors of OS. Median OS was longer for patients with heart V5 ≤ 94.3% (24.7 vs. 16.3 months, *p* = 0.0025), heart V10 ≤ 86.4% (24.8 vs. 16.9 months, *p* = 0.0041), and heart V20 ≤ 76.9% (20.0 vs. 17.2 months, *p* = 0.047). Lower cumulative incidence of symptomatic cardiac adverse events was observed among patients with heart V5 ≤ 94.3% (*p* = 0.017), heart V10 ≤ 86.4% (*p* = 0.02), and heart V20 ≤ 76.9% (*p* = 0.0057). Patients without symptomatic cardiac adverse events had a higher 3-year OS rate (33.8% vs. 0%, *p* = 0.03).

**Conclusions:**

Cardiac radiation dose inversely correlated with survival in ESCC after definitive CCRT. Radiation dose to the heart should be minimized.

## Introduction

Esophageal cancer is the sixth leading cause of cancer-related death globally [1]. Definitive concurrent chemoradiotherapy (CCRT) without surgery is one of treatment options for locally advanced esophageal cancer [2–4]. As the outcome of esophageal cancer after definitive CCRT was unsatisfactory, it is important to find the prognostic factors and improve the treatment.

Radiation dose to the heart was a prognostic factor of non-small cell lung cancer treated with definitive CCRT [5–7]. Similarly, cardiac radiation dose was shown to correlate with overall survival (OS) in a large group of esophageal cancer patients undergoing CCRT with or without surgery [8]. However, the prognostic significance of cardiac radiation dose remains to be elucidated specifically in esophageal cancer after definitive CCRT without surgery.

In the present study, we analyzed a single-institution cohort of esophageal squamous cell carcinoma (ESCC) patients receiving definitive CCRT with intensity modulated radiotherapy (IMRT) technique. The association between cardiac dose-volume parameters and survival was examined.

## Methods

### Patients and study design

This study was approved by the institutional review board of our hospital. Patients with primary ESCC treated by definitive CCRT at our institution between 2008 and 2018 were retrospectively reviewed. The study patient flow diagram was shown in the Additional file 1: Fig. S1. Patients were recruited on the basis of criteria as follows: newly pathologically confirmed ESCC without distant metastasis, no past history of thoracic radiotherapy, CCRT via IMRT and conventional fractionation with dose ≥50 Gy, and follow-up after CCRT ≥3 months. The cases with distant metastasis, past history of thoracic radiotherapy, radiotherapy alone, two- or three-dimensional radiotherapy, radiation dose < 50 Gy, or follow-up < 3 months were excluded. The pre-treatment evaluation of esophageal cancer included esophagogastroduodenoscopy, endoscopic ultrasonography, computed tomography (CT) of the chest and abdomen, and bone scan. Positron emission tomography-computed tomography was performed in cases with indeterminate results of CT or bone scan. The clinical stage was classified according to the seventh edition of the American Joint Committee on Cancer staging system.

### Definitive concurrent chemoradiotherapy

All patients received definitive CCRT for esophageal cancer with IMRT technique, as previously described [9]. Briefly, the gross tumor volume (GTV) consisted of GTV of the primary (GTVp) and GTV of lymph nodes (GTVn). The clinical target volume (CTV) 1 included GTVp with a 5-cm craniocaudal and 1-cm radial margin along the esophagus, and GTVn with a 1-cm margin. The CTV 2 included GTVp with a 2-cm craniocaudal and 1-cm radial margin along the esophagus, and GTVn with a 1-cm margin. The planning target volume (PTV) was generated by expanding 1 cm around the GTV and CTV in all directions. Elective nodal irradiation was omitted. CTV 1 and 2 with the relevant PTV were sequentially treated to 36 and 50–50.4 Gy, respectively. Thereafter, GTV with the relevant PTV was boosted up to 66.6 Gy if dose constraints of the organs at risk could be met. Normal tissue dose constraints included Dmax < 50 Gy for spinal cord, V50 < 33% for heart, V20 < 33% for lung, Dmax < 55 Gy for stomach, and V35 < 50% for liver. During radiation treatment, concurrent chemotherapy and supportive therapy were given.

### Dosimetric analysis

The organs at risk were delineated on each axial slice of simulation CT scan [10, 11]. For heart, the superior aspect began from the level of the inferior border of the pulmonary artery passing the midline and extended inferiorly to the cardiac apex. Dose volume histogram of organs at risk was subsequently generated using the treatment planning system. We calculated the following dose-volume parameters of the heart and lung: mean dose and the percent volumes receiving doses ≥5 Gy (V5), ≥ 10 Gy (V10), ≥ 20 Gy (V20), ≥ 30 Gy (V30), ≥ 40 Gy (V40), and ≥ 50 Gy (V50).

### Evaluation of symptomatic cardiac adverse events

Follow-up evaluations included clinical examinations, esophagogastroduodenoscopy, and CT scan of the chest and abdomen at 1 month after CCRT and then every 3–6 months. In addition, electrocardiography, echocardiogram, and other cardiovascular evaluations were arranged as clinically indicated. To identify symptomatic cardiac adverse events, clinical symptoms and signs, CT images, electrocardiograms, echocardiograms, and managements for cardiovascular diseases were reviewed.

### Statistical analysis

The data cutoff date was June 26, 2019. OS was calculated from the start of IMRT to the date of death or last follow-up. The time to cardiac adverse events was defined as the interval from the beginning of IMRT to the occurrence of events. Survival of patients and cumulative incidence of cardiac events were estimated by the Kaplan-Meier method and compared between groups by the log-rank test. The factors associated with OS were checked with univariate analysis. The independent prognostic factors of OS were examined by multivariate Cox proportional hazards regression analyses in 42 models in which clinical variables with a trend in univariate analysis (*p* < 0.1), one cardiac dose-volume parameter, and one pulmonary parameter were taken into consideration. A *p*-value < 0.05 was considered statistically significant. Statistical analyses were performed with SPSS version 22.0 software and R version 3.5.1 for Windows.

## Results

### Patients’ characteristics

Of the 204 patients reviewed, 121 patients matched the recruitment criteria while 83 patients were excluded from the analysis with reasons as follows: stage IV (*n* = 21), radiation dose < 50 Gy (*n* = 23), post-CCRT follow-up < 3 months (*n* = 32), histology other than squamous cell carcinoma (*n* = 5), and use of 3-dimensional conformal radiotherapy (n = 23). Table 1 summarized demographic and clinical characteristics of the 121 patients, including five women and 116 men. Upper or middle esophagus was involved by the tumor in 104 (86.0%) patients. Furthermore, six (5.0%) patients had a history of cardiovascular diseases (1 coronary artery disease, 3 congestive heart failure, 1 aortic valve infectious endocarditis after valve replacement, and 1 arrhythmia) at baseline. The six patients were considered medically fit for CCRT under the suggestion of our institutional multidisciplinary esophageal cancer team.

**Table 1:** Demographic and clinical characteristics of patients at baseline

Characteristic	No. of patients (%)
Age (years)	
Median (Range)	56 (34–81)
≤ 56	61 (50.4)
> 56	60 (49.6)
Gender	
Male	116 (95.9)
Female	5 (4.1)
Body mass index (kg/m^2^)	
Median (Range)	21.3 (15.5–30.0)
≤ 21.3	61 (50.4)
> 21.3	60 (49.6)
Body surface area (m^2^)	
Median (Range)	1.65 (1.3–2.1)
≤ 1.65	63 (52.1)
> 1.65	58 (47.9)
Eastern Cooperative Oncology Group performance status	
0	11 (9.1)
1	95 (78.5)
2	14 (11.6)
3	1 (0.8)
Stage	
I	2 (1.7)
II	8 (6.6)
III	111 (91.7)
Tumor location	
U	51 (42.1)
M	30 (24.8)
L	17 (14.0)
U + M (from U to M)	9 (7.4)
U + M + L (from U to L)	1 (0.8)
M + L (from M to L)	13 (10.7)
Smoking	
Yes	109 (90.1)
No	12 (9.9)
Alcohol	
Yes	111 (91.7)
No	10 (8.3)
Hypertension	
Yes	24 (19.8)
No	97 (80.2)
Diabetes	
Yes	15 (12.4)
No	106 (87.6)
Cardiovascular disease	
Yes	6 (5.0)
No	115 (95.0)
Heart volume (ml)	
≤ 592	61 (50.4)
> 592	60 (49.6)
Chemotherapy regimen	
Fluoropyrimidine-based	113 (93.4)
Taxane-based	4 (3.3)
Others	4 (3.3)
Radiation dose (Gy)	
Median (range)	61.2 (50–66.6)
≤ 61.2	68 (56.2)
> 61.2	53 (43.8)
PTV prescribed to 36 Gy (ml)	
Median (Range)	780.4 (97.1–1799.5)
PTV prescribed to 50 Gy (ml)	
Median (Range)	640.0 (26.0–1761.2)

Abbreviations: *L* Lower thoracic esophagus, *M* Middle thoracic esophagus, *PTV* Planning target volume, *U* Upper thoracic and cervical esophagus

### Treatment

The utilized chemotherapy regimens and radiation doses were summarized in the Additional file 2: Table S1. The median radiation dose was 61.2 Gy (range, 50–66.6 Gy). The radiation doses were not different between patients with or without pre-existing cardiovascular diseases (*p* = 0.613). Fluoropyrimidine-based chemotherapy regimens were used in 113 (93.4%) patients. Most patients received either cisplatin (25 mg/m^2^) plus fluorouracil (1000 mg/m^2^) given intravenously every week or cisplatin (20 mg/m^2^ daily, on day 1–4) plus fluorouracil (800 mg/m^2^ daily, on day 1–4) given intravenously every 4 weeks. Other regimens were utilized at the discretion of physicians. Furthermore, during CCRT, enteral nutrition support was given via nasogastric, percutaneous endoscopic gastrostomy, and feeding jejunostomy tubes in eight (6.6%), 11 (9.1%), and 17 (14.0%) patients, respectively. Medications for emesis or pain as well as intravenous hydration were given as clinically indicated.

### Clinical characteristics associated with overall survival

**Fig. 1:**
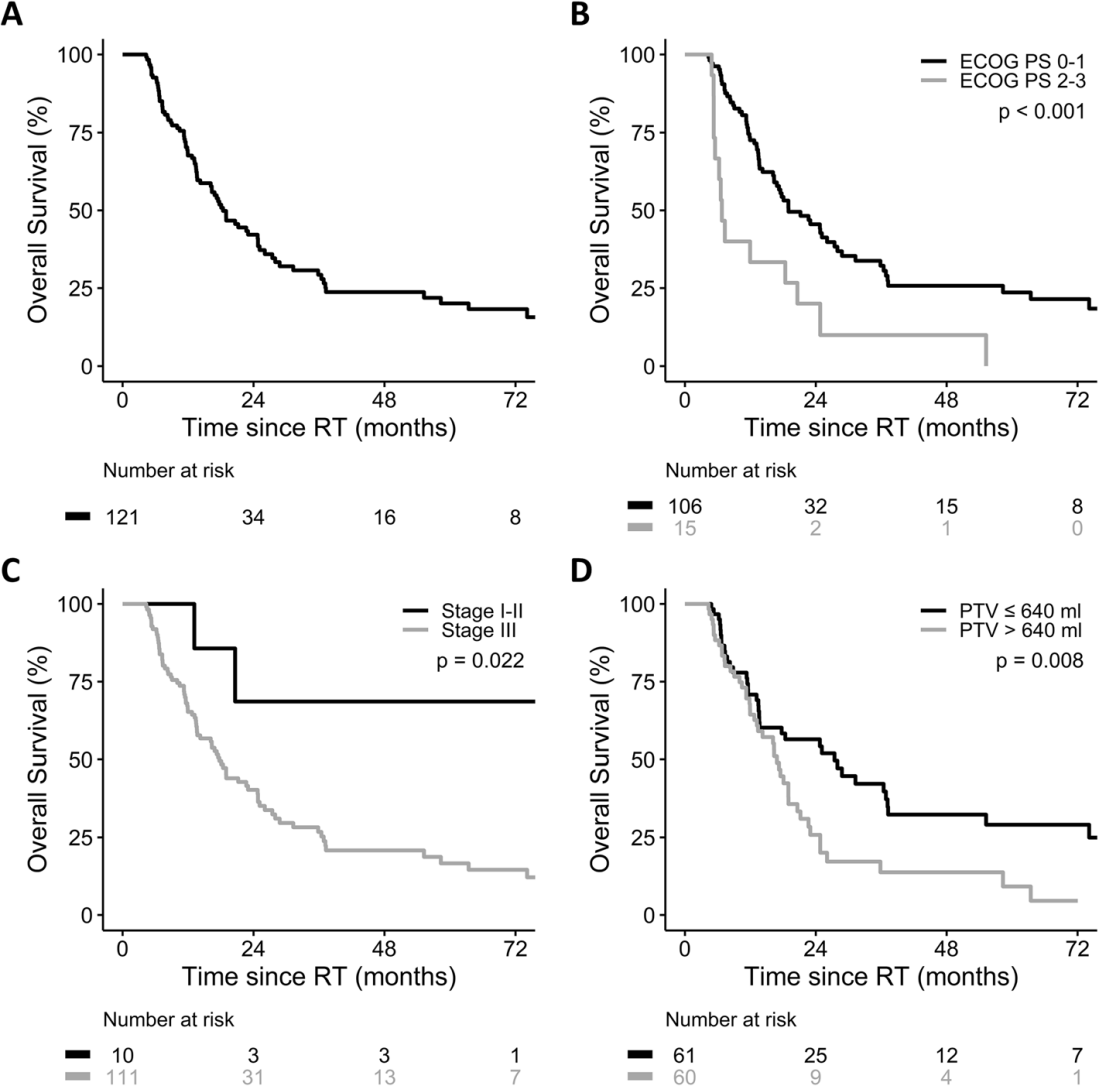
Overall survival **a** whole cohort, **b** by ECOG performance status, **c** by stage, and **d** by volume of PTV prescribed to 50 Gy

The median follow-up was 16.2 (range, 4.3–109.3) and 21.2 months (range, 5.8–104.6) for the whole cohort and the surviving patients, respectively. The median OS of the whole cohort was 18.4 months (Fig. 1a). In univariate analysis, body mass index, body surface area, Eastern Cooperative Oncology Group (ECOG) performance status, stage, chemotherapy regimens, and the volume of PTV prescribed to 50 Gy were associated with OS, but the pre-existing cardiovascular diseases was not correlated with OS (*p* = 0.87, Table 2). Moreover, ECOG performance status, stage, chemotherapy regimens, and the volume of PTV prescribed to 36 Gy and 50 Gy were independent prognostic factors of OS by multivariate analysis (Additional files 3, 4, 5, 6, 7, 8 and 9: Table S2–8)*.* The median OS was longer for patients with ECOG performance status 0–1 (19.0 vs. 6.8 months, *p* < 0.001; Fig. 1b), stage I-II (not reached vs. 17.7 months, *p* = 0.022; Fig. 1c), fluoropyrimidine-based chemotherapy (19.0 vs. 11.2 months, *p* = 0.0016), and volume of PTV prescribed to 50 Gy ≤ 640 ml (27.4 vs. 16.9 months, *p* = 0.008; Fig. 1d). On the other hand, the median volume of overlap between PTV and the heart was 25.1 ml. The median OS was not statistically different between patients with the overlapping volume > 25.1 ml and ≤ 25.1 ml (18.4 vs. 18.1 months, *p* = 0.192).

**Table 2:** Univariate analysis of clinical variables associated with overall survival

Variable	Univariate analysis	
	HR (95% CI)	*P* value
Age (≤ 56 vs. > 56)	0.986 (0.639–1.522)	0.951
Gender (female vs. male)	0.710 (0.224–2.254)	0.561
Body mass index (kg/m^2^) (≤ 21.3 vs. > 21.3)	1.699 (1.096–2.634)	0.018
Body surface area (m^2^) (≤1.65 vs. > 1.65)	1.614 (1.042–2.502)	0.032
ECOG performance status (0–1 vs. 2–3)	0.376 (0.210–0.672)	0.001
Stage (I&II vs. III)	0.223 (0.055–0.910)	0.036
Tumor location (U involved vs. others)	0.733 (0.473–1.136)	0.165
Smoking (no vs. yes)	0.744 (0.358–1.547)	0.428
Alcohol (no vs. yes)	0.529 (0.214–1.310)	0.169
Hypertension (no vs. yes)	1.002 (0.587–1.710)	0.994
Diabetes (no vs. yes)	0.624 (0.335–1.160)	0.136
Cardiovascular disease (no vs. yes)	0.919 (0.336–2.518)	0.870
Heart volume (ml) (≤592 vs > 592)	1.468 (0.947–2.276)	0.086
Chemotherapy regimen (F vs. NF)	0.318 (0.150–0.673)	0.003
Radiation dose (Gy) (≤ 61.2 vs. > 61.2)	1.451 (0.924–2.280)	0.106
PTV prescribed to 36 Gy (ml) (continuous)	1.001 (1.000–1.001)	0.055
PTV prescribed to 50 Gy (ml) (continuous)	1.001 (1.000–1.002)	0.004

Abbreviations: *ECOG* Eastern Cooperative Oncology Group, *F* Fluoropyrimidine-based, *NF* Not fluoropyrimidine-based, *PTV* Planning target volume, *U* Upper thoracic and cervical esophagus

### Dose-volume parameters associated with overall survival

**Fig. 2:**
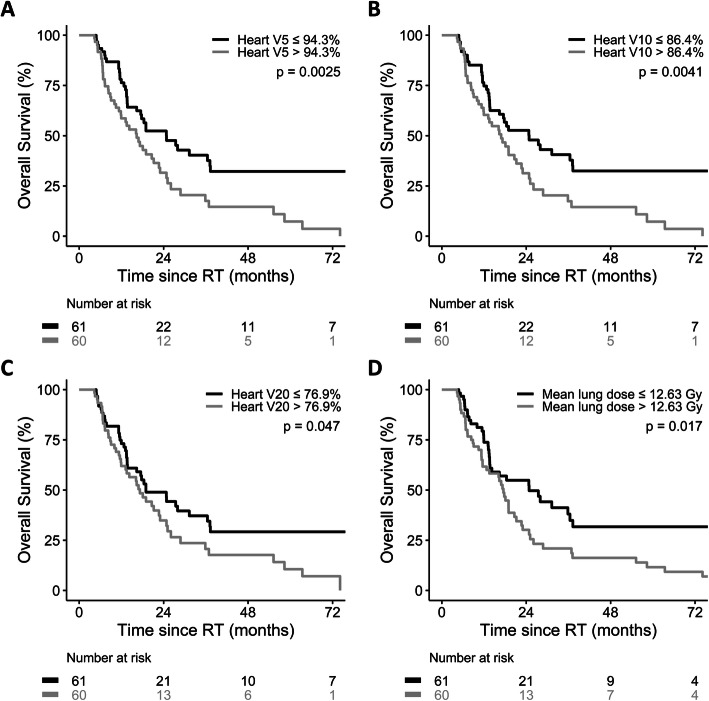
Overall survival by **a** heart V5, **b** heart V10, **c** heart V20, and **d** mean lung dose

Heart V5, V10, and V20 consistently served as independent prognostic factors of OS under consideration of individual pulmonary dose-volume parameters (Table 3 and Additional file 3, 4, 5, 6, 7, 8 and 9: Table S2–8). The median heart V5, V10, and V20 were 94.3, 86.4, and 76.9%, respectively. A longer median OS was observed among patients with heart V5 ≤ 94.3% (24.7 vs. 16.3 months, *p* = 0.0025; Fig. 2a), heart V10 ≤ 86.4% (24.8 vs. 16.9 months, *p* = 0.0041; Fig. 2b), and heart V20 ≤ 76.9% (19.0 vs. 17.2 months, *p* = 0.047; Fig. 2c). In addition, mean lung dose was consistently shown to be a prognostic factor of OS in analytic models including different cardiac dose-volume parameters (Additional file 3, 4, 5, 6, 7, 8, 9 and 10: Table S2–9). Patients with mean lung dose ≤12.63 Gy had a superior median OS (24.8 vs. 17.5 months, *p* = 0.017; Fig. 2d).

**Table 3:** Multivariate analysis for heart dose-volume parameters and overall survival under consideration of different lung parameters

Heart	Mean lung dose	Lung V5	Lung V10	Lung V20	Lung V30	Lung V40
	HR (95% CI) P value					
Mean dose	1.000	1.000	1.000	1.000	1.000	1.000
	(1.000–1.000)	(1.000–1.000)	(1.000–1.000)	(1.000–1.000)	(1.000–1.000)	(1.000–1.000)
	0.052	0.068	0.034	0.035	0.022	0.020
V5	1.011	1.011	1.012	1.012	1.012	1.012
	(1.001–1.020)	(1.001–1.021)	(1.002–1.022)	(1.002–1.021)	(1.003–1.022)	(1.003–1.022)
	0.029	0.032	0.016	0.016	0.010	0.009
V10	1.010	1.010	1.011	1.011	1.011	1.012
	(1.001–1.019)	(1.001–1.020)	(1.002–1.020)	(1.002–1.020)	(1.003–1.020)	(1.003–1.021)
	0.032	0.039	0.020	0.020	0.012	0.010
V20	1.010	1.010	1.011	1.011	1.011	1.012
	(1.001–1.019)	(1.001–1.020)	(1.002–1.020)	(1.002–1.020)	(1.003–1.020)	(1.003–1.021)
	0.029	0.038	0.019	0.020	0.011	0.009
V30	1.010	1.009	1.011	1.010	1.011	1.011
	(1.000–1.020)	(0.999–1.020)	(1.001–1.020)	(1.001–1.020)	(1.002–1.021)	(1.002–1.021)
	0.048	0.068	0.035	0.036	0.021	0.016
V40	1.011	1.011	1.012	1.012	1.012	1.013
	(1.001–1.022)	(1.000–1.022)	(1.001–1.023)	(1.001–1.022)	(1.002–1.023)	(1.002–1.023)
	0.034	0.053	0.028	0.028	0.019	0.017
V50	1.014	1.012	1.014	1.014	1.014	1.014
	(1.000–1.027)	(0.999–1.026)	(1.001–1.028)	(1.001–1.028)	(1.001–1.028)	(1.001–1.028)
	0.046	0.077	0.042	0.038	0.035	0.040

### Dose-volume parameters associated with symptomatic cardiac adverse events

**Fig. 3:**
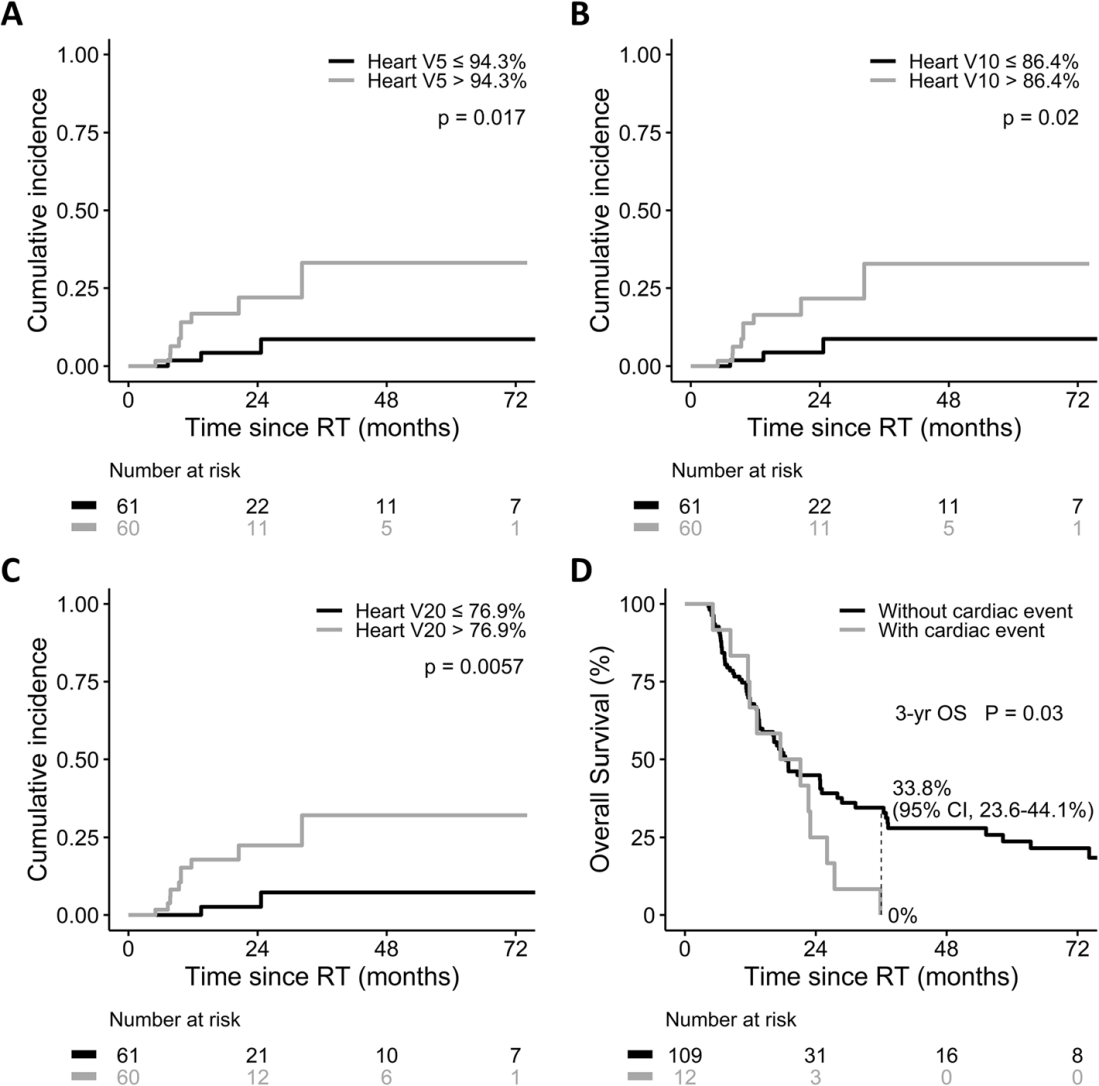
Cumulative incidence of symptomatic cardiac adverse events by **a** heart V5, **b** heart V10, and **c** heart V20. **d** Overall survival by symptomatic cardiac adverse events

There were 12 symptomatic CCRT-related cardiac adverse events, including ischemic heart disease in one, arrhythmia in three, and pericardial effusion in eight patients. The median interval from the start of IMRT to development of cardiac events was 9.8 months. The pre-existing cardiovascular diseases were not associated with the cumulative incidence of cardiac adverse events after CCRT (*p* = 0.459). Lower cumulative incidence of symptomatic cardiac adverse events was found among patients with heart V5 ≤ 94.3% (*p* = 0.017; Fig. 3a), heart V10 ≤ 86.4% (*p* = 0.02; Fig. 3b), and heart V20 ≤ 76.9% (*p* = 0.0057; Fig. 3c). Moreover, patients without symptomatic cardiac adverse events had a higher 3-year OS rate (33.8% vs. 0%, *p* = 0.03; Fig. 3d). There was a trend toward better survival at 2 years in patients without symptomatic cardiac complications (44.3% vs. 25.0%, *p* = 0.23).

### Cancer-specific survival and cause of death

The causes of deaths were shown in Table 4. Fifty-eight (47.9%) patients died of esophageal cancer with or without other etiologies. The median esophageal cancer-specific survival was 24.7 months. As shown in the Additional file 11: Fig. S2, a longer median esophageal cancer-specific survival was observed among patients with heart V5 ≤ 94.3% (36.4 vs. 21.2 months, *p* = 0.042), heart V10 ≤ 86.4% (36.4 vs. 19.0 months, *p* = 0.032), and volume of PTV prescribed to 50 Gy ≤ 640 ml (37.2 vs. 19.0 months, *p* = 0.005). In addition, there was a trend toward better esophageal cancer-specific survival in cases with heart V20 ≤ 76.9% (*p* = 0.2). But the association between the tumor location and esophageal cancer-specific survival was not found (*p* = 0.67).

**Table 4:** Cause of death

Cause of death	No. of deaths (%) (*n* = 83)
Cases with symptomatic cardiac adverse events (*n* = 12)	
Disease progression + Cardiac event + Infection	1 (1.2)
Disease progression + Cardiac event	3 (3.6)
Disease progression + Infection	2 (2.4)
Disease progression	3 (3.6)
Cardiac event	1 (1.2)
Infection	1 (1.2)
Second primary malignancy	1 (1.2)
Cases without symptomatic cardiac adverse events (*n* = 71)	
Disease progression	32 (38.6)
Disease progression + Infection	15 (18.1)
Disease progression + Second primary malignancy	2 (2.4)
Infection	15 (18.1)
Second primary malignancy	6 (7.2)
Unknown	1 (1.2)

## Discussion

The present study analyzed 121 ESCC patients undergoing definitive CCRT with IMRT technique. We identified heart V5, V10, V20, and mean lung dose as independent predictors of prognosis by multivariate analysis. Cardiac radiation dose was correlated with the incidence of symptomatic cardiac adverse events which were inversely associated with survival.

Cardiac dose-volume parameters were found to be independent prognostic factors in a large group of esophageal cancer patients undergoing CCRT [8]. Notably, several key factors differentiated our data from the previously published one. To begin with, the present study specifically analyzed patients treated with definitive CCRT without surgery while the previous research included patients receiving CCRT with or without surgery. In addition, patients with adenocarcinoma and ESCC were combined for investigation in the previous report. The current cohort only included patients with ESCC. To the best of authors’ knowledge, we were the first to show the prognostic significance of cardiac dose-volume parameters in ESCC after definitive CCRT with IMRT technique. Finally, we systematically performed the multivariate analyses with models incorporating different lung and heart dose-volume parameters. The prognostic significance of the cardiac dose was consistently confirmed under individual consideration of different lung dose-volume parameters in the present study.

In line with the large comprehensive research evaluating the association of cardiac dosimetric factors and the outcomes in esophageal cancer after CCRT with or without surgery [8], heart V20 was shown to influence the survival of ESCC treated with definitive CCRT in the present study. However, heart V5 and V10 which acted as independent prognostic factors in the current cohort were not found to independently influence OS in the previously published report. These discrepant results were possibly derived from the different cancer histologies and treatments between studies as the current cohort only reviewed ESCC patients receiving definitive CCRT without surgery while the previous research included patients with adenocarcinoma and ESCC treated by CCRT with or without operation [8]. In addition, heart V30, V40, and V50 were not reported as independent predictors of OS in the present study because the statistical significance was not achieved in multivariate analytic models in which lung V5 was included. But the existing trend suggested that this negative result might be attributed to the limited sample size. Further investigations with expanded cases are warranted in the future. Moreover, the current study found the inferior survival in patients with large PTV which was a surrogate marker of the tumor burden. This data coincided with the evidence that high tumor burden was associated with poor prognosis in cancers treated with definitive CCRT [12–15].

The previous research did not find the association between cardiac radiation dose and survival in the subset analysis of esophageal cancer patients undergoing definitive CCRT [8]. Notably, the present study showed that lower cardiac radiation dose predicted the superior survival in ESCC treated by definitive CCRT. Our finding is novel and needs external validation in independent cohorts. On the other hand, radiation-related heart disease is not only a well-known late effect of low dose irradiation in non-cancer subjects, but also a recognized adverse event of radiotherapy in cancer patients [16]. Symptomatic cardiotoxicity has been reported in esophageal cancer patients undergoing definitive CCRT. High cardiac radiation dose was identified as a risk factor of cardiac complications [9, 17–19]. In the current study, the prevalence of symptomatic cardiac adverse events was 9.92% which was comparable to 5.88–18.97% reported in the literature. Consistent with prior researches of lung and esophageal cancer treated with CCRT [6, 8], we found patients receiving higher radiation dose to the heart had more symptomatic cardiac adverse events which were in turn correlated with a worse survival. The inferior survival might partly result from the mortality which was directly caused by the cardiac complications. Furthermore, the cardiotoxicity which existed after CCRT possibly reduced patients’ tolerance to the salvage treatment upon cancer progression and thereby could result in the unfavorable outcomes. Collectively, our data supported the causal relationship between cardiac radiation dose, cardiac adverse events, and poor survival among ESCC patients receiving definitive CCRT and indicated the importance of sparing the heart from radiation.

Our study was limited by its retrospective research design and all potential inherent biases. In addition, we did dosimetric analyses based on planning CT scan without consideration of cardiac physiological motion. Errors of estimation would exist under such circumstance. Although cardiac synchronized radiotherapy has been developed [20], it is not routinely used in CCRT for esophageal cancer. Better protection of the heart and more precise estimation of cardiac radiation dose would be possible with cardiac synchronized radiotherapy in the future. Moreover, the present study evaluated the dose-volume parameters of the whole heart. The prognostic significance of radiation dose to specific cardiac chambers remains an interesting issue to be investigated in esophageal cancer after CCRT. Finally, the present study only included ESCC patients undergoing definitive CCRT without surgery. The results could not be generalized to patients diagnosed with adenocarcinoma or treated by neoadjuvant CCRT plus operation. But on the positive side, we provided a specific information for ESCC patients receiving definitive CCRT. Further validation with independent cohorts is warranted.

## Conclusions

We were the first to report that the high cardiac radiation dose predicted the inferior outcome of ESCC patients undergoing definitive concurrent chemotherapy and IMRT. Radiation dose to the heart should be minimized.

## Supplementary information

**Additional file 1: Figure S1.** Study patient flow diagram.

**Additional file 2: Table S1.** Summary of the Chemotherapy Regimens and Radiation Doses.

**Additional file 3: Table S2.** Multivariate Analysis for Mean Heart Dose and Overall Survival.

**Additional file 4: Table S3.** Multivariate Analysis for Heart V5 and Overall Survival.

**Additional file 5: Table S4.** Multivariate Analysis for Heart V10 and Overall Survival.

**Additional file 6: Table S5.** Multivariate Analysis for Heart V20 and Overall Survival.

**Additional file 7: Table S6.** Multivariate Analysis for Heart V30 and Overall Survival.

**Additional file 8: Table S7.** Multivariate Analysis for Heart V40 and Overall Survival.

**Additional file 9: Table S8.** Multivariate Analysis for Heart V50 and Overall Survival.

**Additional file 10: Table S9.** Multivariate Analysis for Lung Dose-volume Parameters and Overall Survival under Consideration of Different Heart Parameters.

**Additional file 11: Figure S2.** Cancer-specific survival by A) heart V5, B) heart V10, C) heart V20, and D) volume of PTV prescribed to 50 Gy.

## Data Availability

The datasets used and/or analyzed during the current study are available from the corresponding author on reasonable request.

## Abbreviations

CCRT: Concurrent chemoradiotherapy
CT: Computed tomography
CTV: Clinical target volume
ECOG: Eastern Cooperative Oncology Group
ESCC: Esophageal squamous cell carcinoma
GTV: Gross tumor volume
GTVn: Gross tumor volume of lymph nodes
GTVp: Gross tumor volume of the primary
IMRT: Intensity modulated radiotherapy
OS: Overall survival
PTV: Planning target volume
