# Supplementary figures and images for: Cardiac radiation dose predicts survival in esophageal squamous cell carcinoma treated by definitive concurrent chemotherapy and intensity modulated radiotherapy
Source: Radiat Oncol. 2020 Sep 22;15:221. doi: 10.1186/s13014-020-01664-7 (PMC7510071; doi:10.1186/s13014-020-01664-7)

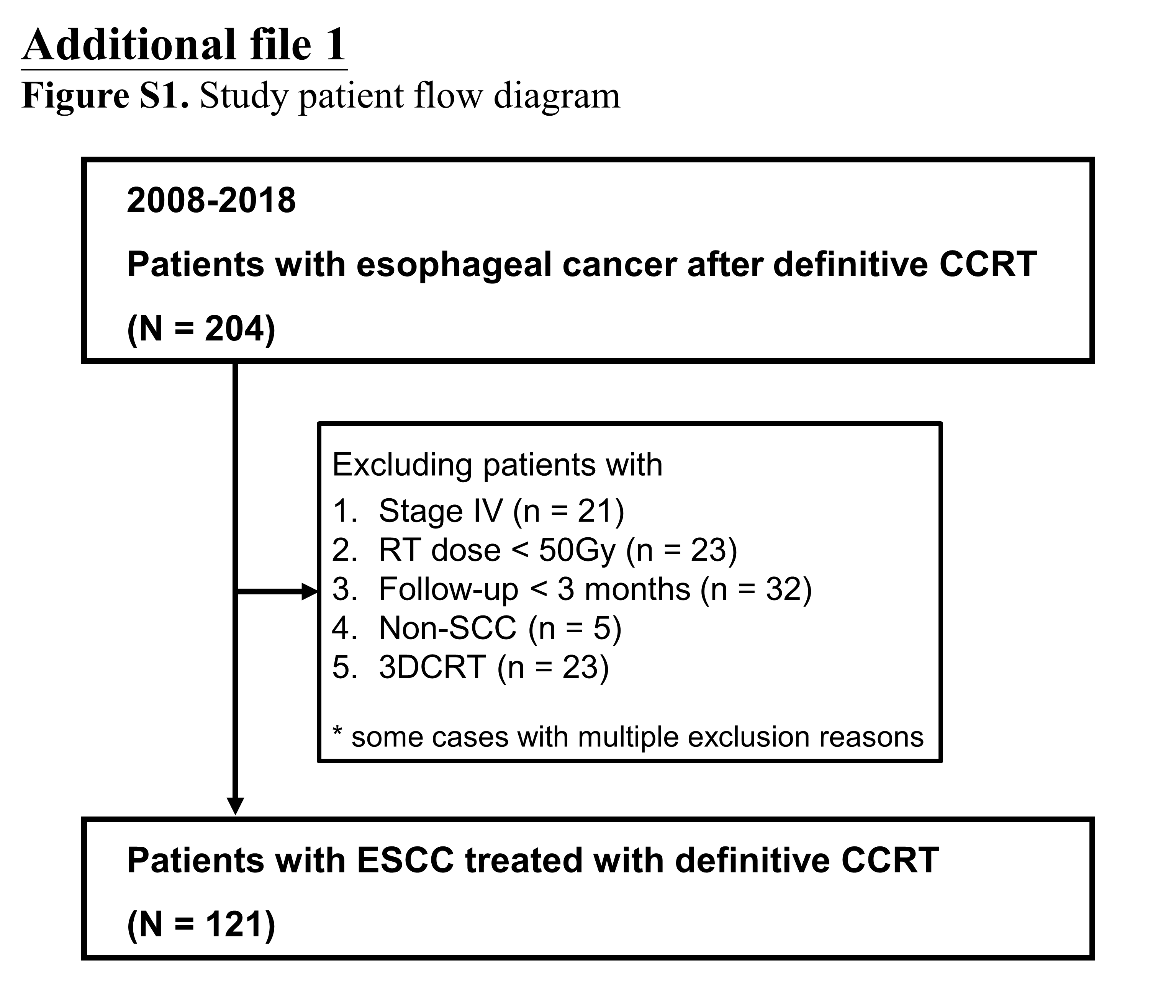

Supplement: Supplementary file 1 — Additional file 1: Figure S1. Study patient flow diagram. [file 13014_2020_1664_MOESM1_ESM.tif]

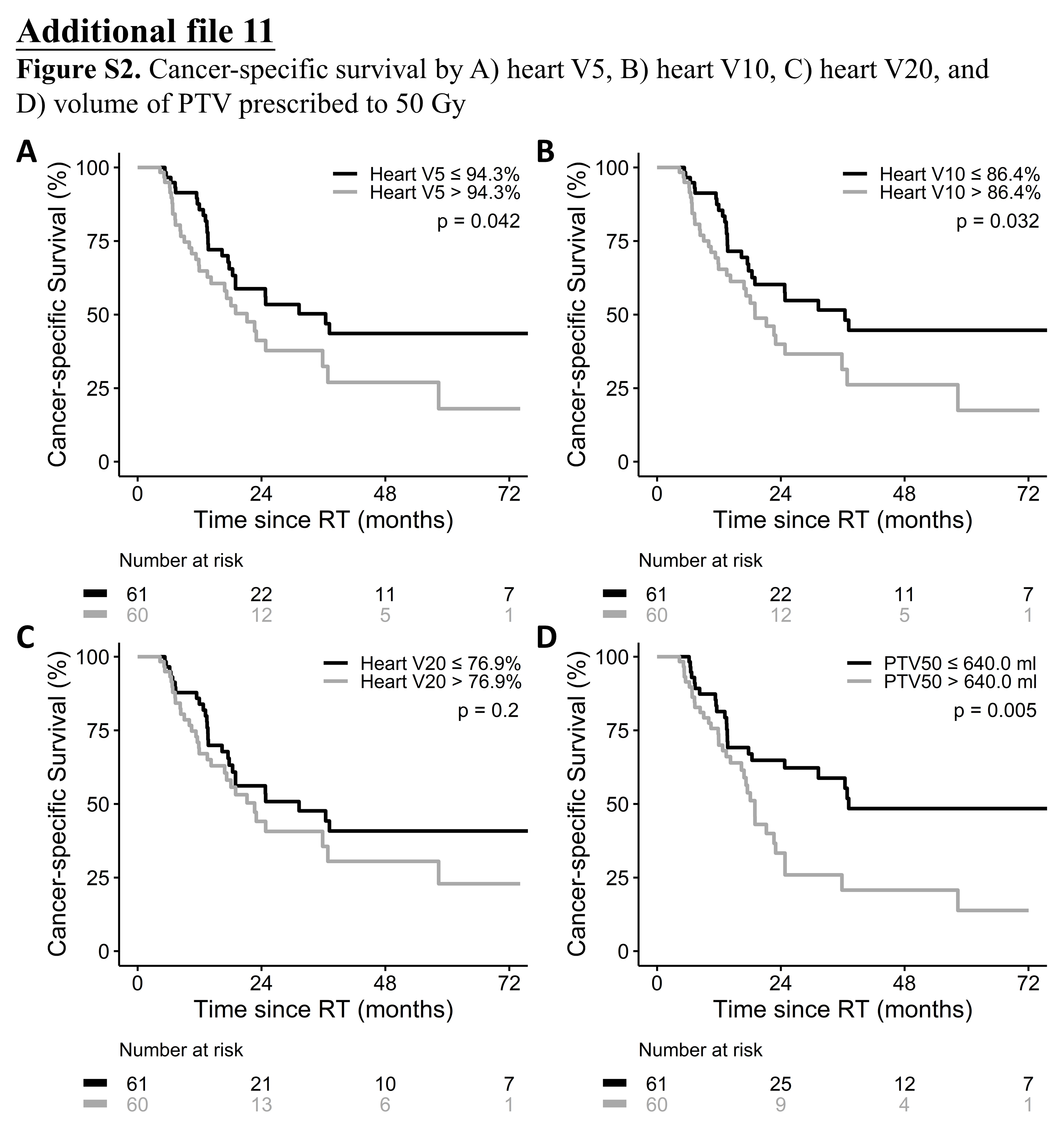

Supplement: Supplementary file 11 — Additional file 11: Figure S2. Cancer-specific survival by A) heart V5, B) heart V10, C) heart V20, and D) volume of PTV prescribed to 50 Gy. [file 13014_2020_1664_MOESM11_ESM.tif]
