# Supplementary material for: Cardiac radiation dose predicts survival in esophageal squamous cell carcinoma treated by definitive concurrent chemotherapy and intensity modulated radiotherapy
Source: Radiat Oncol. 2020 Sep 22;15:221. doi: 10.1186/s13014-020-01664-7 (PMC7510071; doi:10.1186/s13014-020-01664-7)
Supplement: Supplementary file 2 — Additional file 2: Table S1. Summary of the Chemotherapy Regimens and Radiation Doses. [file 13014_2020_1664_MOESM2_ESM.pdf]

Additional file 2

Table S1. Summary of the Chemotherapy Regimens and Radiation Doses

**Chemotherapy regimen and radiation dose**	**Number (%) (n = 121)**
**Cases with pre-existing cardiovascular disease (n = 6)**	
**Chemotherapy regimen**	
P (25 mg/m^2) + F (1000 mg/m^2) QW	3 (2.5)
F (2000 mg/m^2) + Carboplatin (AUC 4) Q2W	1 (0.8)
T (50 mg/m^2) + Carboplatin (AUC 2) QW	1 (0.8)
P (40 mg/m^2) QW	1 (0.8)
**Radiation Dose (Gy)**	
Median (Range)	61.2 (50-66.6)
**Cases without pre-existing cardiovascular disease (n = 115)**	
**Chemotherapy regimen**	
P (25 mg/m^2) + F (1000 mg/m^2) QW	56 (46.3)
P (20 mg/m^2 daily, D1–4) + F (800 mg/m^2 daily, D1–4) Q4W	31 (25.6)
P (25-40 mg/m^2) + F (2000-2600 mg/m^2) + L (200-300 mg/m^2) Q2W	9 (7.4)
P (20-25 mg/m^2) + F (1500-2000 mg/m^2) + L (150-200 mg/m^2) QW	9 (7.4)
P (25 mg/m^2) + F (2000 mg/m^2) + L (200 mg/m^2) + Cet (400 → 250 mg/m^2) QW	1 (0.8)
P (20 mg/m^2) + F (1600 mg/m^2) + L (160 mg/m^2) + E (30 mg/m^2) QW	1 (0.8)
F (1600 mg/m^2) QW	1 (0.8)
F (1600 mg/m^2) + L (160 mg/m^2) QW	1 (0.8)
T (80 mg/m^2) + P (15 mg/m^2) QW	2 (1.7)
T (35 mg/m^2) + P (15 mg/m^2) + Cet (400 → 250 mg/m^2) QW	1 (0.8)
P (40 mg/m^2) QW	1 (0.8)
Docetaxel (35 mg/m2) + P (35 mg/m^2) + F (1500 mg/m^2) Q2W	1 (0.8)
T (110 mg/m^2) + P (40 mg/m^2) + F (3000 mg/m^2) + L (400 mg/m^2) Q2W	1 (0.8)
**Radiation Dose (Gy)**	
Median (Range)	61.2 (50-66.6)

Abbreviations: *Cet* cetuximab, *D* day, *E* etoposide, *F* fluorouracil, *L* leucovorin, *P* cisplatin, *QW* weekly, *Q2W* every 2 weeks, *Q4W* every 4 weeks, *T* paclitaxel
