# Supplementary material for: Cardiac radiation dose predicts survival in esophageal squamous cell carcinoma treated by definitive concurrent chemotherapy and intensity modulated radiotherapy
Source: Radiat Oncol. 2020 Sep 22;15:221. doi: 10.1186/s13014-020-01664-7 (PMC7510071; doi:10.1186/s13014-020-01664-7)
Supplement: Supplementary file 3 — Additional file 3: Table S2. Multivariate Analysis for Mean Heart Dose and Overall Survival. [file 13014_2020_1664_MOESM3_ESM.pdf]

Additional file 3

Table S2. Multivariate Analysis for Mean Heart Dose and Overall Survival

	Variables	HR	95% CI	*P* value
**Model Mean heart dose/ Mean lung dose**	Body mass index (kg/m^2) (≤ 21.3 vs. > 21.3)	1.747	0.889-3.432	0.105
	Body surface area (m^2) (≤1.65 vs. > 1.65)	1.179	0.589-2.363	0.642
	ECOG performance status (0–1 vs. 2–3)	0.235	0.118-0.472	0.000
	Stage (I&II vs. III)	0.026	0.004-0.190	0.000
	Heart volume (ml) (≤592 vs. >592)	0.691	0.414-1.153	0.157
	Chemotherapy regimen (F vs. NF)	0.175	0.067-0.455	0.000
	PTV prescribed to 36 Gy (ml) (continuous)	0.997	0.996-0.999	0.003
	PTV prescribed to 50 Gy (ml) (continuous)	1.003	1.001-1.005	0.000
	**Mean heart dose (cGy) (continuous)**	1.000	1.000-1.000	0.052
	**Mean lung dose (cGy) (continuous)**	1.001	1.000-1.002	0.017
**Model Mean heart dose/ Lung V5**	Body mass index (kg/m^2) (≤ 21.3 vs. > 21.3)	1.542	0.796-2.990	0.199
	Body surface area (m^2) (≤1.65 vs. > 1.65)	1.454	0.749-2.824	0.269
	ECOG performance status (0–1 vs. 2–3)	0.267	0.136-0.524	0.000
	Stage (I&II vs. III)	0.047	0.008-0.289	0.001
	Heart volume (ml) (≤592 vs. >592)	0.720	0.431-1.203	0.210
	Chemotherapy regimen (F vs. NF)	0.233	0.094-0.579	0.002
	PTV prescribed to 36 Gy (ml) (continuous)	0.998	0.997-1.000	0.016
	PTV prescribed to 50 Gy (ml) (continuous)	1.002	1.001-1.004	0.003
	**Mean heart dose (cGy) (continuous)**	1.000	1.000-1.000	0.068
	**Lung V5 (%) (continuous)**	1.017	0.990-1.044	0.222
**Model Mean heart dose/ Lung V10**	Body mass index (kg/m^2) (≤ 21.3 vs. > 21.3)	1.498	0.776-2.890	0.229
	Body surface area (m^2) (≤1.65 vs. > 1.65)	1.458	0.751-2.831	0.265
	ECOG performance status (0–1 vs. 2–3)	0.257	0.130-0.509	0.000
	Stage (I&II vs. III)	0.046	0.008-0.281	0.001
	Heart volume (ml) (≤592 vs. >592)	0.705	0.421-1.183	0.186
	Chemotherapy regimen (F vs. NF)	0.237	0.095-0.593	0.002
	PTV prescribed to 36 Gy (ml) (continuous)	0.998	0.997-1.000	0.019
	PTV prescribed to 50 Gy (ml) (continuous)	1.002	1.001-1.004	0.003
	**Mean heart dose (cGy) (continuous)**	1.000	1.000-1.000	0.034
	**Lung V10 (%) (continuous)**	1.017	0.981-1.055	0.354

**Model Mean heart dose/ Lung V20**	Body mass index (kg/m^2) (≤ 21.3 vs. > 21.3)	1.484	0.772-2.853	0.236
	Body surface area (m^2) (≤1.65 vs. > 1.65)	1.383	0.710-2.694	0.341
	ECOG performance status (0–1 vs. 2–3)	0.233	0.114-0.473	0.000
	Stage (I&II vs. III)	0.040	0.006-0.256	0.001
	Heart volume (ml) (≤592 vs. >592)	0.677	0.402-1.141	0.143
	Chemotherapy regimen (F vs. NF)	0.224	0.089-0.566	0.002
	PTV prescribed to 36 Gy (ml) (continuous)	0.998	0.996-1.000	0.012
	PTV prescribed to 50 Gy (ml) (continuous)	1.003	1.001-1.004	0.002
	**Mean heart dose (cGy) (continuous)**	1.000	1.000-1.000	0.035
	**Lung V20 (%) (continuous)**	1.037	0.984-1.092	0.171
**Model Mean heart dose/ Lung V30**	Body mass index (kg/m^2) (≤ 21.3 vs. > 21.3)	1.499	0.777-2.891	0.227
	Body surface area (m^2) (≤1.65 vs. > 1.65)	1.390	0.710-2.718	0.337
	ECOG performance status (0–1 vs. 2–3)	0.233	0.115-0.474	0.000
	Stage (I&II vs. III)	0.040	0.006-0.250	0.001
	Heart volume (ml) (≤592 vs. >592)	0.667	0.393-1.130	0.132
	Chemotherapy regimen (F vs. NF)	0.240	0.097-0.594	0.002
	PTV prescribed to 36 Gy (ml) (continuous)	0.998	0.996-1.000	0.013
	PTV prescribed to 50 Gy (ml) (continuous)	1.003	1.001-1.004	0.002
	**Mean heart dose (cGy) (continuous)**	1.000	1.000-1.000	0.022
	**Lung V30 (%) (continuous)**	1.042	0.980-1.107	0.190
**Model Mean heart dose/ Lung V40**	Body mass index (kg/m^2) (≤ 21.3 vs. > 21.3)	1.476	0.763-2.855	0.248
	Body surface area (m^2) (≤1.65 vs. > 1.65)	1.441	0.738-2.811	0.284
	ECOG performance status (0–1 vs. 2–3)	0.245	0.122-0.491	0.000
	Stage (I&II vs. III)	0.041	0.007-0.254	0.001
	Heart volume (ml) (≤592 vs. >592)	0.676	0.398-1.148	0.147
	Chemotherapy regimen (F vs. NF)	0.262	0.108-0.636	0.003
	PTV prescribed to 36 Gy (ml) (continuous)	0.998	0.996-1.000	0.017
	PTV prescribed to 50 Gy (ml) (continuous)	1.003	1.001-1.004	0.002
	**Mean heart dose (cGy) (continuous)**	1.000	1.000-1.000	0.020
	**Lung V40 (%) (continuous)**	1.040	0.970-1.115	0.273

Abbreviations: *ECOG* Eastern Cooperative Oncology Group, *F* fluoropyrimidine-based, *NF* not fluoropyrimidine-based, *PTV* planning target volume, *Vx* percentage of the heart volume receiving more than x gray
