# Supplementary material for: Cardiac radiation dose predicts survival in esophageal squamous cell carcinoma treated by definitive concurrent chemotherapy and intensity modulated radiotherapy
Source: Radiat Oncol. 2020 Sep 22;15:221. doi: 10.1186/s13014-020-01664-7 (PMC7510071; doi:10.1186/s13014-020-01664-7)
Supplement: Supplementary file 4 — Additional file 4: Table S3. Multivariate Analysis for Heart V5 and Overall Survival. [file 13014_2020_1664_MOESM4_ESM.pdf]

Additional file 4

Table S3. Multivariate Analysis for Heart V5 and Overall Survival

Model	Variables	HR	95% CI	*P* value
**Model Heart V5/ Mean lung dose**	Body mass index (kg/m^2) (≤ 21.3 vs. > 21.3)	1.742	0.891-3.407	0.105
	Body surface area (m^2) (≤1.65 vs. > 1.65)	1.160	0.584-2.306	0.672
	ECOG performance status (0–1 vs. 2–3)	0.240	0.120-0.479	0.000
	Stage (I&II vs. III)	0.029	0.004-0.208	0.000
	Chemotherapy regimen (F vs. NF)	0.164	0.063-0.427	0.000
	Heart volume (ml) (≤592 vs. >592)	0.723	0.439-1.191	0.203
	PTV prescribed to 36 Gy (ml) (continuous)	0.998	0.996-0.999	0.005
	PTV prescribed to 50 Gy (ml) (continuous)	1.003	1.001-1.004	0.001
	**Heart V5 (%) (continuous)**	1.011	1.001-1.020	0.029
	**Mean lung dose (cGy) (continuous)**	1.001	1.000-1.002	0.024
**Model Heart V5/ Lung V5**	Body mass index (kg/m^2) (≤ 21.3 vs. > 21.3)	1.547	0.801-2.991	0.194
	Body surface area (m^2) (≤1.65 vs. > 1.65)	1.410	0.731-2.722	0.306
	ECOG performance status (0–1 vs. 2–3)	0.273	0.140-0.533	0.000
	Stage (I&II vs. III)	0.050	0.008-0.307	0.001
	Chemotherapy regimen (F vs. NF)	0.215	0.087-0.532	0.001
	Heart volume (ml) (≤592 vs. >592)	0.763	0.464-1.256	0.288
	PTV prescribed to 36 Gy (ml) (continuous)	0.998	0.997-1.000	0.025
	PTV prescribed to 50 Gy (ml) (continuous)	1.002	1.001-1.004	0.005
	**Heart V5 (%) (continuous)**	1.011	1.001-1.021	0.032
	**Lung V5 (%) (continuous)**	1.015	0.988-1.042	0.291
**Model Heart V5/ Lung V10**	Body mass index (kg/m^2) (≤ 21.3 vs. > 21.3)	1.512	0.785-2.910	0.216
	Body surface area (m^2) (≤1.65 vs. > 1.65)	1.406	0.729-2.712	0.309
	ECOG performance status (0–1 vs. 2–3)	0.263	0.133-0.517	0.000
	Stage (I&II vs. III)	0.049	0.008-0.300	0.001
	Chemotherapy regimen (F vs. NF)	0.216	0.086-0.539	0.001
	Heart volume (ml) (≤592 vs. >592)	0.753	0.454-1.247	0.270
	PTV prescribed to 36 Gy (ml) (continuous)	0.998	0.997-1.000	0.030
	PTV prescribed to 50 Gy (ml) (continuous)	1.002	1.001-1.004	0.005
	**Heart V5 (%) (continuous)**	1.012	1.002-1.022	0.016
	**Lung V10 (%) (continuous)**	1.016	0.979-1.055	0.406

**Model Heart V5/ Lung V20**	Body mass index (kg/m^2) (≤ 21.3 vs. > 21.3)	1.506	0.785-2.888	0.218
	Body surface area (m^2) (≤1.65 vs. > 1.65)	1.333	0.688-2.581	0.394
	ECOG performance status (0–1 vs. 2–3)	0.237	0.117-0.481	0.000
	Stage (I&II vs. III)	0.043	0.007-0.273	0.001
	Chemotherapy regimen (F vs. NF)	0.204	0.081-0.514	0.001
	Heart volume (ml) (≤592 vs. >592)	0.716	0.429-1.196	0.202
	PTV prescribed to 36 Gy (ml) (continuous)	0.998	0.996-1.000	0.018
	PTV prescribed to 50 Gy (ml) (continuous)	1.002	1.001-1.004	0.003
	**Heart V5 (%) (continuous)**	1.012	1.002-1.021	0.016
	**Lung V20 (%) (continuous)**	1.036	0.982-1.092	0.195
**Model Heart V5/ Lung V30**	Body mass index (kg/m^2) (≤ 21.3 vs. > 21.3)	1.522	0.791-2.928	0.208
	Body surface area (m^2) (≤1.65 vs. > 1.65)	1.330	0.684-2.584	0.401
	ECOG performance status (0–1 vs. 2–3)	0.236	0.116-0.478	0.000
	Stage (I&II vs. III)	0.042	0.007-0.265	0.001
	Chemotherapy regimen (F vs. NF)	0.215	0.087-0.531	0.001
	Heart volume (ml) (≤592 vs. >592)	0.702	0.418-1.179	0.182
	PTV prescribed to 36 Gy (ml) (continuous)	0.998	0.996-1.000	0.018
	PTV prescribed to 50 Gy (ml) (continuous)	1.003	1.001-1.004	0.003
	**Heart V5 (%) (continuous)**	1.012	1.003-1.022	0.010
	**Lung V30 (%) (continuous)**	1.044	0.980-1.111	0.180
**Model Heart V5/ Lung V40**	Body mass index (kg/m^2) (≤ 21.3 vs. > 21.3)	1.498	0.776-2.889	0.228
	Body surface area (m^2) (≤1.65 vs. > 1.65)	1.374	0.709-2.664	0.347
	ECOG performance status (0–1 vs. 2–3)	0.249	0.125-0.496	0.000
	Stage (I&II vs. III)	0.043	0.007-0.270	0.001
	Chemotherapy regimen (F vs. NF)	0.234	0.096-0.569	0.001
	Heart volume (ml) (≤592 vs. >592)	0.713	0.424-1.201	0.204
	PTV prescribed to 36 Gy (ml) (continuous)	0.998	0.996-1.000	0.023
	PTV prescribed to 50 Gy (ml) (continuous)	1.003	1.001-1.004	0.004
	**Heart V5 (%) (continuous)**	1.012	1.003-1.022	0.009
	**Lung V40 (%) (continuous)**	1.043	0.971-1.120	0.249

Abbreviations: *ECOG* Eastern Cooperative Oncology Group, *F* fluoropyrimidine-based, *NF* not fluoropyrimidine-based, *PTV* planning target volume, *Vx* percentage of the heart volume receiving more than x gray
