# Supplementary material for: Cardiac radiation dose predicts survival in esophageal squamous cell carcinoma treated by definitive concurrent chemotherapy and intensity modulated radiotherapy
Source: Radiat Oncol. 2020 Sep 22;15:221. doi: 10.1186/s13014-020-01664-7 (PMC7510071; doi:10.1186/s13014-020-01664-7)
Supplement: Supplementary file 5 — Additional file 5: Table S4. Multivariate Analysis for Heart V10 and Overall Survival. [file 13014_2020_1664_MOESM5_ESM.pdf]

Additional file 5

Table S4. Multivariate Analysis for Heart V10 and Overall Survival

	Variables	HR	95% CI	*P* value
**Model Heart V10/ Mean lung dose**	Body mass index (kg/m^2) (≤ 21.3 vs. > 21.3)	1.744	0.890-3.417	0.105
	Body surface area (m^2) (≤1.65 vs. > 1.65)	1.155	0.580-2.299	0.682
	ECOG performance status (0–1 vs. 2–3)	0.240	0.120-0.479	0.000
	Stage (I&II vs. III)	0.029	0.004-0.209	0.000
	Chemotherapy regimen (F vs. NF)	0.165	0.064-0.429	0.000
	Heart volume (ml) (≤592 vs. >592)	0.720	0.436-1.188	0.198
	PTV prescribed to 36 Gy (ml) (continuous)	0.998	0.996-0.999	0.004
	PTV prescribed to 50 Gy (ml) (continuous)	1.003	1.001-1.005	0.001
	**Heart V10 (%) (continuous)**	1.010	1.001-1.019	0.032
	**Mean lung dose (cGy) (continuous)**	1.001	1.000-1.002	0.021
**Model Heart V10/ Lung V5**	Body mass index (kg/m^2) (≤ 21.3 vs. > 21.3)	1.545	0.798-2.990	0.197
	Body surface area (m^2) (≤1.65 vs. > 1.65)	1.410	0.729-2.726	0.307
	ECOG performance status (0–1 vs. 2–3)	0.271	0.139-0.532	0.000
	Stage (I&II vs. III)	0.051	0.008-0.310	0.001
	Chemotherapy regimen (F vs. NF)	0.217	0.088-0.538	0.001
	Heart volume (ml) (≤592 vs. >592)	0.757	0.459-1.248	0.275
	PTV prescribed to 36 Gy (ml) (continuous)	0.998	0.997-1.000	0.022
	PTV prescribed to 50 Gy (ml) (continuous)	1.002	1.001-1.004	0.004
	**Heart V10 (%) (continuous)**	1.010	1.001-1.020	0.039
	**Lung V5 (%) (continuous)**	1.015	0.988-1.043	0.268
**Model Heart V10/ Lung V10**	Body mass index (kg/m^2) (≤ 21.3 vs. > 21.3)	1.506	0.781-2.903	0.221
	Body surface area (m^2) (≤1.65 vs. > 1.65)	1.407	0.728-2.719	0.309
	ECOG performance status (0–1 vs. 2–3)	0.262	0.133-0.517	0.000
	Stage (I&II vs. III)	0.050	0.008-0.304	0.001
	Chemotherapy regimen (F vs. NF)	0.219	0.088-0.546	0.001
	Heart volume (ml) (≤592 vs. >592)	0.747	0.450-1.238	0.258
	PTV prescribed to 36 Gy (ml) (continuous)	0.998	0.997-1.000	0.028
	PTV prescribed to 50 Gy (ml) (continuous)	1.002	1.001-1.004	0.004
	**Heart V10 (%) (continuous)**	1.011	1.002-1.020	0.020
	**Lung V10 (%) (continuous)**	1.016	0.979-1.055	0.398

**Model Heart V10/ Lung V20**	Body mass index (kg/m^2) (≤ 21.3 vs. > 21.3)	1.498	0.780-2.877	0.225
	Body surface area (m^2) (≤1.65 vs. > 1.65)	1.335	0.688-2.588	0.393
	ECOG performance status (0–1 vs. 2–3)	0.237	0.117-0.481	0.000
	Stage (I&II vs. III)	0.044	0.007-0.277	0.001
	Chemotherapy regimen (F vs. NF)	0.207	0.082-0.521	0.001
	Heart volume (ml) (≤592 vs. >592)	0.713	0.426-1.191	0.196
	PTV prescribed to 36 Gy (ml) (continuous)	0.998	0.996-1.000	0.017
	PTV prescribed to 50 Gy (ml) (continuous)	1.003	1.001-1.004	0.003
	**Heart V10 (%) (continuous)**	1.011	1.002-1.020	0.020
	**Lung V20 (%) (continuous)**	1.036	0.983-1.092	0.191
**Model Heart V10/ Lung V30**	Body mass index (kg/m^2) (≤ 21.3 vs. > 21.3)	1.515	0.787-2.918	0.214
	Body surface area (m^2) (≤1.65 vs. > 1.65)	1.332	0.684-2.591	0.399
	ECOG performance status (0–1 vs. 2–3)	0.236	0.116-0.478	0.000
	Stage (I&II vs. III)	0.043	0.007-0.269	0.001
	Chemotherapy regimen (F vs. NF)	0.219	0.088-0.540	0.001
	Heart volume (ml) (≤592 vs. >592)	0.698	0.415-1.173	0.175
	PTV prescribed to 36 Gy (ml) (continuous)	0.998	0.996-1.000	0.016
	PTV prescribed to 50 Gy (ml) (continuous)	1.003	1.001-1.004	0.002
	**Heart V10 (%) (continuous)**	1.011	1.003-1.020	0.012
	**Lung V30 (%) (continuous)**	1.044	0.981-1.110	0.178
**Model Heart V10/ Lung V40**	Body mass index (kg/m^2) (≤ 21.3 vs. > 21.3)	1.491	0.772-2.879	0.235
	Body surface area (m^2) (≤1.65 vs. > 1.65)	1.376	0.709-2.671	0.345
	ECOG performance status (0–1 vs. 2–3)	0.248	0.124-0.494	0.000
	Stage (I&II vs. III)	0.044	0.007-0.272	0.001
	Chemotherapy regimen (F vs. NF)	0.238	0.097-0.579	0.002
	Heart volume (ml) (≤592 vs. >592)	0.706	0.419-1.191	0.192
	PTV prescribed to 36 Gy (ml) (continuous)	0.998	0.996-1.000	0.021
	PTV prescribed to 50 Gy (ml) (continuous)	1.003	1.001-1.004	0.003
	**Heart V10 (%) (continuous)**	1.012	1.003-1.021	0.010
	**Lung V40 (%) (continuous)**	1.044	0.972-1.121	0.236

Abbreviations: *ECOG* Eastern Cooperative Oncology Group, *F* fluoropyrimidine-based, *NF* not fluoropyrimidine-based, *PTV* planning target volume, *Vx* percentage of the heart volume receiving more than x gray
