# Supplementary material for: Cardiac radiation dose predicts survival in esophageal squamous cell carcinoma treated by definitive concurrent chemotherapy and intensity modulated radiotherapy
Source: Radiat Oncol. 2020 Sep 22;15:221. doi: 10.1186/s13014-020-01664-7 (PMC7510071; doi:10.1186/s13014-020-01664-7)
Supplement: Supplementary file 6 — Additional file 6: Table S5. Multivariate Analysis for Heart V20 and Overall Survival. [file 13014_2020_1664_MOESM6_ESM.pdf]

Additional file 6

Table S5. Multivariate Analysis for Heart V20 and Overall Survival

	Variables	HR	95% CI	*P* value
**Model Heart V20/ Mean lung dose**	Body mass index (kg/m^2) (≤ 21.3 vs. > 21.3)	1.748	0.889-3.437	0.105
	Body surface area (m^2) (≤1.65 vs. > 1.65)	1.167	0.584-2.330	0.662
	ECOG performance status (0–1 vs. 2–3)	0.237	0.119-0.476	0.000
	Stage (I&II vs. III)	0.029	0.004-0.208	0.000
	Chemotherapy regimen (F vs. NF)	0.168	0.065-0.434	0.000
	Heart volume (ml) (≤592 vs. >592)	0.709	0.428-1.174	0.181
	PTV prescribed to 36 Gy (ml) (continuous)	0.997	0.996-0.999	0.004
	PTV prescribed to 50 Gy (ml) (continuous)	1.003	1.001-1.005	0.001
	**Heart V20 (%) (continuous)**	1.010	1.001-1.019	0.029
	**Mean lung dose (cGy) (continuous)**	1.001	1.000-1.002	0.021
**Model Heart V20/ Lung V5**	Body mass index (kg/m^2) (≤ 21.3 vs. > 21.3)	1.545	0.796-3.000	0.198
	Body surface area (m^2) (≤1.65 vs. > 1.65)	1.423	0.734-2.761	0.296
	ECOG performance status (0–1 vs. 2–3)	0.266	0.135-0.524	0.000
	Stage (I&II vs. III)	0.050	0.008-0.306	0.001
	Chemotherapy regimen (F vs. NF)	0.220	0.089-0.544	0.001
	Heart volume (ml) (≤592 vs. >592)	0.740	0.446-1.226	0.243
	PTV prescribed to 36 Gy (ml) (continuous)	0.998	0.997-1.000	0.019
	PTV prescribed to 50 Gy (ml) (continuous)	1.002	1.001-1.004	0.004
	**Heart V20 (%) (continuous)**	1.010	1.001-1.020	0.038
	**Lung V5 (%) (continuous)**	1.015	0.988-1.042	0.280
**Model Heart V20/ Lung V10**	Body mass index (kg/m^2) (≤ 21.3 vs. > 21.3)	1.507	0.779-2.915	0.223
	Body surface area (m^2) (≤1.65 vs. > 1.65)	1.425	0.735-2.764	0.295
	ECOG performance status (0–1 vs. 2–3)	0.258	0.130-0.512	0.000
	Stage (I&II vs. III)	0.050	0.008-0.301	0.001
	Chemotherapy regimen (F vs. NF)	0.223	0.090-0.555	0.001
	Heart volume (ml) (≤592 vs. >592)	0.731	0.439-1.218	0.229
	PTV prescribed to 36 Gy (ml) (continuous)	0.998	0.997-1.000	0.025
	PTV prescribed to 50 Gy (ml) (continuous)	1.002	1.001-1.004	0.004
	**Heart V20 (%) (continuous)**	1.011	1.002-1.020	0.019
	**Lung V10 (%) (continuous)**	1.015	0.978-1.053	0.436

**Model Heart V20/ Lung V20**	Body mass index (kg/m^2) (≤ 21.3 vs. > 21.3)	1.497	0.776-2.885	0.229
	Body surface area (m^2) (≤1.65 vs. > 1.65)	1.357	0.697-2.641	0.370
	ECOG performance status (0–1 vs. 2–3)	0.236	0.116-0.480	0.000
	Stage (I&II vs. III)	0.044	0.007-0.278	0.001
	Chemotherapy regimen (F vs. NF)	0.211	0.084-0.531	0.001
	Heart volume (ml) (≤592 vs. >592)	0.703	0.419-1.177	0.180
	PTV prescribed to 36 Gy (ml) (continuous)	0.998	0.996-1.000	0.016
	PTV prescribed to 50 Gy (ml) (continuous)	1.003	1.001-1.004	0.003
	**Heart V20 (%) (continuous)**	1.011	1.002-1.020	0.020
	**Lung V20 (%) (continuous)**	1.034	0.980-1.090	0.221
**Model Heart V20/ Lung V30**	Body mass index (kg/m^2) (≤ 21.3 vs. > 21.3)	1.513	0.783-2.924	0.218
	Body surface area (m^2) (≤1.65 vs. > 1.65)	1.354	0.693-2.642	0.375
	ECOG performance status (0–1 vs. 2–3)	0.234	0.115-0.475	0.000
	Stage (I&II vs. III)	0.043	0.007-0.269	0.001
	Chemotherapy regimen (F vs. NF)	0.222	0.090-0.548	0.001
	Heart volume (ml) (≤592 vs. >592)	0.687	0.408-1.157	0.158
	PTV prescribed to 36 Gy (ml) (continuous)	0.998	0.996-1.000	0.015
	PTV prescribed to 50 Gy (ml) (continuous)	1.003	1.001-1.004	0.002
	**Heart V20 (%) (continuous)**	1.011	1.003-1.020	0.011
	**Lung V30 (%) (continuous)**	1.041	0.979-1.107	0.198
**Model Heart V20/ Lung V40**	Body mass index (kg/m^2) (≤ 21.3 vs. > 21.3)	1.489	0.769-2.884	0.238
	Body surface area (m^2) (≤1.65 vs. > 1.65)	1.395	0.717-2.715	0.327
	ECOG performance status (0–1 vs. 2–3)	0.244	0.122-0.488	0.000
	Stage (I&II vs. III)	0.043	0.007-0.269	0.001
	Chemotherapy regimen (F vs. NF)	0.240	0.099-0.584	0.002
	Heart volume (ml) (≤592 vs. >592)	0.691	0.409-1.168	0.168
	PTV prescribed to 36 Gy (ml) (continuous)	0.998	0.996-1.000	0.017
	PTV prescribed to 50 Gy (ml) (continuous)	1.003	1.001-1.004	0.003
	**Heart V20 (%) (continuous)**	1.012	1.003-1.021	0.009
	**Lung V40 (%) (continuous)**	1.043	0.973-1.119	0.236

Abbreviations: *ECOG* Eastern Cooperative Oncology Group, *F* fluoropyrimidine-based, *NF* not fluoropyrimidine-based, *PTV* planning target volume, *Vx* percentage of the heart volume receiving more than x gray
