# Supplementary material for: Cardiac radiation dose predicts survival in esophageal squamous cell carcinoma treated by definitive concurrent chemotherapy and intensity modulated radiotherapy
Source: Radiat Oncol. 2020 Sep 22;15:221. doi: 10.1186/s13014-020-01664-7 (PMC7510071; doi:10.1186/s13014-020-01664-7)
Supplement: Supplementary file 7 — Additional file 7: Table S6. Multivariate Analysis for Heart V30 and Overall Survival. [file 13014_2020_1664_MOESM7_ESM.pdf]

Additional file 7

Table S6. Multivariate Analysis for Heart V30 and Overall Survival

	Variables	HR	95% CI	*P* value
**Model Heart V30/ Mean lung dose**	Body mass index (kg/m^2) (≤ 21.3 vs. > 21.3)	1.757	0.893-3.456	0.102
	Body surface area (m^2) (≤1.65 vs. > 1.65)	1.171	0.585-2.340	0.656
	ECOG performance status (0–1 vs. 2–3)	0.229	0.114-0.459	0.000
	Stage (I&II vs. III)	0.029	0.004-0.205	0.000
	Chemotherapy regimen (F vs. NF)	0.170	0.066-0.440	0.000
	Heart volume (ml) (≤592 vs. >592)	0.696	0.417-1.161	0.166
	PTV prescribed to 36 Gy (ml) (continuous)	0.997	0.996-0.999	0.004
	PTV prescribed to 50 Gy (ml) (continuous)	1.003	1.001-1.004	0.001
	**Heart V30 (%) (continuous)**	1.010	1.000-1.020	0.048
	**Mean lung dose (cGy) (continuous)**	1.001	1.000-1.002	0.017
**Model Heart V30/ Lung V5**	Body mass index (kg/m^2) (≤ 21.3 vs. > 21.3)	1.556	0.801-3.021	0.192
	Body surface area (m^2) (≤1.65 vs. > 1.65)	1.433	0.738-2.781	0.288
	ECOG performance status (0–1 vs. 2–3)	0.257	0.130-0.508	0.000
	Stage (I&II vs. III)	0.050	0.008-0.303	0.001
	Chemotherapy regimen (F vs. NF)	0.224	0.090-0.553	0.001
	Heart volume (ml) (≤592 vs. >592)	0.724	0.433-1.211	0.218
	PTV prescribed to 36 Gy (ml) (continuous)	0.998	0.997-1.000	0.019
	PTV prescribed to 50 Gy (ml) (continuous)	1.002	1.001-1.004	0.005
	**Heart V30 (%) (continuous)**	1.009	0.999-1.020	0.068
	**Lung V5 (%) (continuous)**	1.016	0.990-1.043	0.230
**Model Heart V30/ Lung V10**	Body mass index (kg/m^2) (≤ 21.3 vs. > 21.3)	1.513	0.782-2.927	0.219
	Body surface area (m^2) (≤1.65 vs. > 1.65)	1.436	0.740-2.787	0.285
	ECOG performance status (0–1 vs. 2–3)	0.248	0.125-0.492	0.000
	Stage (I&II vs. III)	0.050	0.008-0.297	0.001
	Chemotherapy regimen (F vs. NF)	0.228	0.092-0.566	0.001
	Heart volume (ml) (≤592 vs. >592)	0.712	0.424-1.195	0.199
	PTV prescribed to 36 Gy (ml) (continuous)	0.998	0.997-1.000	0.025
	PTV prescribed to 50 Gy (ml) (continuous)	1.002	1.001-1.004	0.005
	**Heart V30 (%) (continuous)**	1.011	1.001-1.020	0.035
	**Lung V10 (%) (continuous)**	1.016	0.980-1.054	0.389

Model Heart V30/ Lung V20	Body mass index (kg/m^2) (≤ 21.3 vs. > 21.3)	1.498	0.778-2.887	0.227
	Body surface area (m^2) (≤1.65 vs. > 1.65)	1.365	0.701-2.658	0.360
	ECOG performance status (0–1 vs. 2–3)	0.226	0.111-0.458	0.000
	Stage (I&II vs. III)	0.044	0.007-0.273	0.001
	Chemotherapy regimen (F vs. NF)	0.215	0.086-0.541	0.001
	Heart volume (ml) (≤592 vs. >592)	0.685	0.407-1.155	0.156
	PTV prescribed to 36 Gy (ml) (continuous)	0.998	0.996-1.000	0.015
	PTV prescribed to 50 Gy (ml) (continuous)	1.002	1.001-1.004	0.003
	**Heart V30 (%) (continuous)**	1.010	1.001-1.020	0.036
	**Lung V20 (%) (continuous)**	1.036	0.983-1.091	0.189
Model Heart V30/ Lung V30	Body mass index (kg/m^2) (≤ 21.3 vs. > 21.3)	1.515	0.784-2.927	0.217
	Body surface area (m^2) (≤1.65 vs. > 1.65)	1.367	0.700-2.670	0.360
	ECOG performance status (0–1 vs. 2–3)	0.225	0.111-0.456	0.000
	Stage (I&II vs. III)	0.043	0.007-0.265	0.001
	Chemotherapy regimen (F vs. NF)	0.229	0.093-0.563	0.001
	Heart volume (ml) (≤592 vs. >592)	0.672	0.397-1.140	0.141
	PTV prescribed to 36 Gy (ml) (continuous)	0.998	0.996-1.000	0.015
	PTV prescribed to 50 Gy (ml) (continuous)	1.003	1.001-1.004	0.003
	**Heart V30 (%) (continuous)**	1.011	1.002-1.021	0.021
	**Lung V30 (%) (continuous)**	1.041	0.980-1.107	0.191
Model Heart V30/ Lung V40	Body mass index (kg/m^2) (≤ 21.3 vs. > 21.3)	1.489	0.768-2.886	0.238
	Body surface area (m^2) (≤1.65 vs. > 1.65)	1.410	0.724-2.745	0.312
	ECOG performance status (0–1 vs. 2–3)	0.234	0.117-0.468	0.000
	Stage (I&II vs. III)	0.043	0.007-0.265	0.001
	Chemotherapy regimen (F vs. NF)	0.247	0.102-0.601	0.002
	Heart volume (ml) (≤592 vs. >592)	0.675	0.397-1.149	0.147
	PTV prescribed to 36 Gy (ml) (continuous)	0.998	0.996-1.000	0.018
	PTV prescribed to 50 Gy (ml) (continuous)	1.002	1.001-1.004	0.004
	**Heart V30 (%) (continuous)**	1.011	1.002-1.021	0.016
	**Lung V40 (%) (continuous)**	1.044	0.974-1.118	0.226

Abbreviations: *ECOG* Eastern Cooperative Oncology Group, *F* fluoropyrimidine-based, *NF* not fluoropyrimidine-based, *PTV* planning target volume, *Vx* percentage of the heart volume receiving more than x gray
