# Supplementary material for: Cardiac radiation dose predicts survival in esophageal squamous cell carcinoma treated by definitive concurrent chemotherapy and intensity modulated radiotherapy
Source: Radiat Oncol. 2020 Sep 22;15:221. doi: 10.1186/s13014-020-01664-7 (PMC7510071; doi:10.1186/s13014-020-01664-7)
Supplement: Supplementary file 8 — Additional file 8: Table S7. Multivariate Analysis for Heart V40 and Overall Survival. [file 13014_2020_1664_MOESM8_ESM.pdf]

Additional file 8

Table S7. Multivariate Analysis for Heart V40 and Overall Survival

	Variables	HR	95% CI	*P* value
**Model Heart V40/ Mean lung dose**	Body mass index (kg/m^2) (≤ 21.3 vs. > 21.3)	1.725	0.883-3.372	0.111
	Body surface area (m^2) (≤1.65 vs. > 1.65)	1.190	0.596-2.374	0.622
	ECOG performance status (0–1 vs. 2–3)	0.226	0.113-0.455	0.000
	Stage (I&II vs. III)	0.025	0.004-0.182	0.000
	Chemotherapy regimen (F vs. NF)	0.174	0.067-0.451	0.000
	Heart volume (ml) (≤592 vs. >592)	0.673	0.400-1.132	0.136
	PTV prescribed to 36 Gy (ml) (continuous)	0.997	0.996-0.999	0.002
	PTV prescribed to 50 Gy (ml) (continuous)	1.003	1.001-1.005	0.000
	**Heart V40 (%) (continuous)**	1.011	1.001-1.022	0.034
	**Mean lung dose (cGy) (continuous)**	1.001	1.000-1.002	0.012
**Model Heart V40/ Lung V5**	Body mass index (kg/m^2) (≤ 21.3 vs. > 21.3)	1.526	0.790-2.946	0.208
	Body surface area (m^2) (≤1.65 vs. > 1.65)	1.467	0.758-2.841	0.255
	ECOG performance status (0–1 vs. 2–3)	0.257	0.130-0.507	0.000
	Stage (I&II vs. III)	0.046	0.008-0.282	0.001
	Chemotherapy regimen (F vs. NF)	0.231	0.093-0.574	0.002
	Heart volume (ml) (≤592 vs. >592)	0.700	0.414-1.182	0.182
	PTV prescribed to 36 Gy (ml) (continuous)	0.998	0.996-1.000	0.013
	PTV prescribed to 50 Gy (ml) (continuous)	1.002	1.001-1.004	0.003
	**Heart V40 (%) (continuous)**	1.011	1.000-1.022	0.053
	**Lung V5 (%) (continuous)**	1.018	0.993-1.044	0.164
**Model Heart V40/ Lung V10**	Body mass index (kg/m^2) (≤ 21.3 vs. > 21.3)	1.474	0.768-2.829	0.244
	Body surface area (m^2) (≤1.65 vs. > 1.65)	1.472	0.761-2.846	0.250
	ECOG performance status (0–1 vs. 2–3)	0.246	0.124-0.489	0.000
	Stage (I&II vs. III)	0.045	0.007-0.274	0.001
	Chemotherapy regimen (F vs. NF)	0.236	0.095-0.589	0.002
	Heart volume (ml) (≤592 vs. >592)	0.685	0.404-1.160	0.159
	PTV prescribed to 36 Gy (ml) (continuous)	0.998	0.996-1.000	0.016
	PTV prescribed to 50 Gy (ml) (continuous)	1.002	1.001-1.004	0.003
	**Heart V40 (%) (continuous)**	1.012	1.001-1.023	0.028
	**Lung V10 (%) (continuous)**	1.019	0.984-1.056	0.286

**Model Heart V40/ Lung V20**	Body mass index (kg/m^2) (≤ 21.3 vs. > 21.3)	1.456	0.762-2.784	0.255
	Body surface area (m^2) (≤1.65 vs. > 1.65)	1.397	0.720-2.709	0.323
	ECOG performance status (0–1 vs. 2–3)	0.223	0.110-0.452	0.000
	Stage (I&II vs. III)	0.039	0.006-0.248	0.001
	Chemotherapy regimen (F vs. NF)	0.224	0.089-0.564	0.002
	Heart volume (ml) (≤592 vs. >592)	0.658	0.387-1.119	0.123
	PTV prescribed to 36 Gy (ml) (continuous)	0.998	0.996-0.999	0.009
	PTV prescribed to 50 Gy (ml) (continuous)	1.003	1.001-1.004	0.002
	**Heart V40 (%) (continuous)**	1.012	1.001-1.022	0.028
	**Lung V20 (%) (continuous)**	1.039	0.988-1.093	0.138
**Model Heart V40/ Lung V30**	Body mass index (kg/m^2) (≤ 21.3 vs. > 21.3)	1.470	0.766-2.821	0.246
	Body surface area (m^2) (≤1.65 vs. > 1.65)	1.406	0.722-2.740	0.316
	ECOG performance status (0–1 vs. 2–3)	0.224	0.110-0.455	0.000
	Stage (I&II vs. III)	0.039	0.006-0.243	0.001
	Chemotherapy regimen (F vs. NF)	0.243	0.099-0.598	0.002
	Heart volume (ml) (≤592 vs. >592)	0.651	0.381-1.111	0.116
	PTV prescribed to 36 Gy (ml) (continuous)	0.998	0.996-0.999	0.011
	PTV prescribed to 50 Gy (ml) (continuous)	1.003	1.001-1.004	0.002
	**Heart V40 (%) (continuous)**	1.012	1.002-1.023	0.019
	**Lung V30 (%) (continuous)**	1.043	0.982-1.107	0.172
**Model Heart V40/ Lung V40**	Body mass index (kg/m^2) (≤ 21.3 vs. > 21.3)	1.446	0.751-2.784	0.270
	Body surface area (m^2) (≤1.65 vs. > 1.65)	1.459	0.751-2.836	0.265
	ECOG performance status (0–1 vs. 2–3)	0.234	0.117-0.470	0.000
	Stage (I&II vs. III)	0.040	0.006-0.245	0.001
	Chemotherapy regimen (F vs. NF)	0.265	0.109-0.642	0.003
	Heart volume (ml) (≤592 vs. >592)	0.657	0.384-1.126	0.127
	PTV prescribed to 36 Gy (ml) (continuous)	0.998	0.996-1.000	0.015
	PTV prescribed to 50 Gy (ml) (continuous)	1.003	1.001-1.004	0.003
	**Heart V40 (%) (continuous)**	1.013	1.002-1.023	0.017
	**Lung V40 (%) (continuous)**	1.042	0.973-1.116	0.235

Abbreviations: *ECOG* Eastern Cooperative Oncology Group, *F* fluoropyrimidine-based, *NF* not fluoropyrimidine-based, *PTV* planning target volume, *Vx* percentage of the heart volume receiving more than x gray
