# Supplementary material for: Cardiac radiation dose predicts survival in esophageal squamous cell carcinoma treated by definitive concurrent chemotherapy and intensity modulated radiotherapy
Source: Radiat Oncol. 2020 Sep 22;15:221. doi: 10.1186/s13014-020-01664-7 (PMC7510071; doi:10.1186/s13014-020-01664-7)
Supplement: Supplementary file 9 — Additional file 9: Table S8. Multivariate Analysis for Heart V50 and Overall Survival. [file 13014_2020_1664_MOESM9_ESM.pdf]

Additional file 9

Table S8. Multivariate Analysis for Heart V50 and Overall Survival

	Variables	HR	95% CI	*P* value
**Model Heart V50/ Mean lung dose**	Body mass index (kg/m^2) (≤ 21.3 vs. > 21.3)	1.737	0.880-3.427	0.111
	Body surface area (m^2) (≤1.65 vs. > 1.65)	1.199	0.590-2.437	0.615
	ECOG performance status (0–1 vs. 2–3)	0.234	0.117-0.469	0.000
	Stage (I&II vs. III)	0.021	0.003-0.155	0.000
	Chemotherapy regimen (F vs. NF)	0.193	0.073-0.508	0.001
	Heart volume (ml) (≤592 vs. >592)	0.660	0.389-1.121	0.124
	PTV prescribed to 36 Gy (ml) (continuous)	0.997	0.995-0.999	0.002
	PTV prescribed to 50 Gy (ml) (continuous)	1.003	1.002-1.005	0.000
	**Heart V50 (%) (continuous)**	1.014	1.000-1.027	0.046
	**Mean lung dose (cGy) (continuous)**	1.001	1.000-1.002	0.009
**Model Heart V50/ Lung V5**	Body mass index (kg/m^2) (≤ 21.3 vs. > 21.3)	1.517	0.780-2.950	0.219
	Body surface area (m^2) (≤1.65 vs. > 1.65)	1.520	0.772-2.991	0.225
	ECOG performance status (0–1 vs. 2–3)	0.269	0.137-0.529	0.000
	Stage (I&II vs. III)	0.041	0.006-0.257	0.001
	Chemotherapy regimen (F vs. NF)	0.264	0.104-0.669	0.005
	Heart volume (ml) (≤592 vs. >592)	0.690	0.404-1.176	0.172
	PTV prescribed to 36 Gy (ml) (continuous)	0.998	0.996-1.000	0.012
	PTV prescribed to 50 Gy (ml) (continuous)	1.003	1.001-1.004	0.002
	**Heart V50 (%) (continuous)**	1.012	0.999-1.026	0.077
	**Lung V5 (%) (continuous)**	1.020	0.995-1.045	0.113
**Model Heart V50/ Lung V10**	Body mass index (kg/m^2) (≤ 21.3 vs. > 21.3)	1.456	0.753-2.814	0.264
	Body surface area (m^2) (≤1.65 vs. > 1.65)	1.528	0.777-3.004	0.219
	ECOG performance status (0–1 vs. 2–3)	0.256	0.129-0.507	0.000
	Stage (I&II vs. III)	0.039	0.006-0.245	0.001
	Chemotherapy regimen (F vs. NF)	0.273	0.107-0.695	0.006
	Heart volume (ml) (≤592 vs. >592)	0.668	0.391-1.143	0.141
	PTV prescribed to 36 Gy (ml) (continuous)	0.998	0.996-1.000	0.014
	PTV prescribed to 50 Gy (ml) (continuous)	1.003	1.001-1.004	0.002
	**Heart V50 (%) (continuous)**	1.014	1.001-1.028	0.042
	**Lung V10 (%) (continuous)**	1.023	0.988-1.058	0.199

**Model Heart V50/ Lung V20**	Body mass index (kg/m²) (≤ 21.3 vs. > 21.3)	1.433	0.745-2.755	0.281
	Body surface area (m²) (≤1.65 vs. > 1.65)	1.440	0.729-2.842	0.294
	ECOG performance status (0–1 vs. 2–3)	0.228	0.113-0.464	0.000
	Stage (I&II vs. III)	0.033	0.005-0.216	0.000
	Chemotherapy regimen (F vs. NF)	0.260	0.101-0.668	0.005
	Heart volume (ml) (≤592 vs. >592)	0.640	0.373-1.099	0.106
	PTV prescribed to 36 Gy (ml) (continuous)	0.998	0.996-0.999	0.008
	PTV prescribed to 50 Gy (ml) (continuous)	1.003	1.001-1.005	0.001
	**Heart V50 (%) (continuous)**	1.014	1.001-1.028	0.038
	**Lung V20 (%) (continuous)**	1.043	0.993-1.096	0.095
**Model Heart V50/ Lung V30**	Body mass index (kg/m²) (≤ 21.3 vs. > 21.3)	1.446	0.748-2.796	0.273
	Body surface area (m²) (≤1.65 vs. > 1.65)	1.456	0.732-2.895	0.284
	ECOG performance status (0–1 vs. 2–3)	0.233	0.115-0.472	0.000
	Stage (I&II vs. III)	0.034	0.005-0.217	0.000
	Chemotherapy regimen (F vs. NF)	0.288	0.115-0.720	0.008
	Heart volume (ml) (≤592 vs. >592)	0.642	0.373-1.105	0.110
	PTV prescribed to 36 Gy (ml) (continuous)	0.998	0.996-0.999	0.011
	PTV prescribed to 50 Gy (ml) (continuous)	1.003	1.001-1.005	0.001
	**Heart V50 (%) (continuous)**	1.014	1.001-1.028	0.035
	**Lung V30 (%) (continuous)**	1.044	0.984-1.107	0.157
**Model Heart V50/ Lung V40**	Body mass index (kg/m²) (≤ 21.3 vs. > 21.3)	1.427	0.734-2.771	0.294
	Body surface area (m²) (≤1.65 vs. > 1.65)	1.520	0.764-3.023	0.232
	ECOG performance status (0–1 vs. 2–3)	0.248	0.124-0.496	0.000
	Stage (I&II vs. III)	0.036	0.006-0.225	0.000
	Chemotherapy regimen (F vs. NF)	0.316	0.129-0.775	0.012
	Heart volume (ml) (≤592 vs. >592)	0.660	0.383-1.135	0.133
	PTV prescribed to 36 Gy (ml) (continuous)	0.998	0.996-1.000	0.018
	PTV prescribed to 50 Gy (ml) (continuous)	1.003	1.001-1.005	0.002
	**Heart V50 (%) (continuous)**	1.014	1.001-1.028	0.040
	**Lung V40 (%) (continuous)**	1.037	0.967-1.111	0.306

Abbreviations: *ECOG* Eastern Cooperative Oncology Group, *F* fluoropyrimidine-based, *NF* not fluoropyrimidine-based, *PTV* planning target volume, *Vx* percentage of the heart volume receiving more than x gray
