# Supplementary material for: Cardiac radiation dose predicts survival in esophageal squamous cell carcinoma treated by definitive concurrent chemotherapy and intensity modulated radiotherapy
Source: Radiat Oncol. 2020 Sep 22;15:221. doi: 10.1186/s13014-020-01664-7 (PMC7510071; doi:10.1186/s13014-020-01664-7)
Supplement: Supplementary file 10 — Additional file 10: Table S9. Multivariate Analysis for Lung Dose-volume Parameters and Overall Survival under Consideration of Different Heart Parameters. [file 13014_2020_1664_MOESM10_ESM.pdf]

Additional file 10

Table S9. Multivariate Analysis for Lung Dose-volume Parameters and Overall Survival under Consideration of Different Heart Parameters

Lung	Mean heart dose	Heart V5	Heart V10	Heart V20	Heart V30	Heart V40	Heart V50
	HR (95% CI) P value						
Mean dose	1.001	1.001	1.001	1.001	1.001	1.001	1.001
	(1.000-1.002)	(1.000-1.002)	(1.000-1.002)	(1.000-1.002)	(1.000-1.002)	(1.000-1.002)	(1.000-1.002)
	0.017	0.024	0.021	0.021	0.017	0.012	0.009
V5	1.017	1.015	1.015	1.015	1.016	1.018	1.020
	(0.990-1.044)	(0.988-1.042)	(0.988-1.043)	(0.988-1.042)	(0.990-1.043)	(0.993-1.044)	(0.995-1.045)
	0.222	0.291	0.268	0.280	0.230	0.164	0.113
V10	1.017	1.016	1.016	1.015	1.016	1.019	1.023
	(0.981-1.055)	(0.979-1.055)	(0.979-1.055)	(0.978-1.053)	(0.980-1.054)	(0.984-1.056)	(0.988-1.058)
	0.354	0.406	0.398	0.436	0.389	0.286	0.199
V20	1.037	1.036	1.036	1.034	1.036	1.039	1.043
	(0.984-1.092)	(0.982-1.092)	(0.983-1.092)	(0.980-1.090)	(0.983-1.091)	(0.988-1.093)	(0.993-1.096)
	0.171	0.195	0.191	0.221	0.189	0.138	0.095
V30	1.042	1.044	1.044	1.041	1.041	1.043	1.044
	(0.980-1.107)	(0.980-1.111)	(0.981-1.110)	(0.979-1.107)	(0.980-1.107)	(0.982-1.107)	(0.984-1.107)
	0.190	0.180	0.178	0.198	0.191	0.172	0.157
V40	1.040	1.043	1.044	1.043	1.044	1.042	1.037
	(0.970-1.115)	(0.971-1.120)	(0.972-1.121)	(0.973-1.119)	(0.974-1.118)	(0.973-1.116)	(0.967-1.111)
	0.273	0.249	0.236	0.236	0.226	0.235	0.306
